# Causal relationship between psychiatric traits and temporomandibular disorders: a bidirectional two-sample Mendelian randomization study

**DOI:** 10.1007/s00784-023-05339-x

**Published:** 2023-11-01

**Authors:** Yulin Xiang, Jukun Song, Ying Liang, Jiaxin Sun, Zhijun Zheng

**Affiliations:** 1 School of Stomatology, Zunyi Medical University, Zunyi, China; 2 Department of Endodontics, Guiyang Stomatological Hospital, 253 Jiefang Road, Nanming District, Guiyang, 550005 Guizhou China; 3 Department of Oral and Maxillofacial Surgery, The Affiliated Stomatological Hospital of Guizhou Medical University, Guiyang, China

**Keywords:** Psychiatric traits, Temporomandibular disorders, Mendelian randomization, Causal relationship

## Abstract

**Objectives:**

This study was to investigate the causal relationship between temporomandibular disorders (TMD) and psychiatric disorders by Mendelian randomization (MR) analysis.

**Materials and methods:**

A two-sample bidirectional MR analysis was adopted to systematically explore the causal relationship between TMD and eight psychiatric traits, including anxiety disorder (AD), panic disorder (PD), major depressive disorder (MDD), neuroticism, attention deficit hyperactivity disorder (ADHD), autism spectrum disorder (ASD), bipolar disorder (BIP), and schizophrenia (SCZ). Inverse variance weighted (IVW), weighted median, and MR-Egger regression were used in my study. Furthermore, we also performed three sensitivity analyses to illustrate the reliability of the analysis.

**Results:**

Two psychiatric traits have risk effects on TMD: PD (OR = 1.118, 95% CI: 1.047–1.194, *P* = 8.161 × 10^−4^, MDD (OR = 1.961, 95% CI: 1.450–2.653, *P* = 1.230 × 10^−5^). Despite not surpassing the strict Bonferroni correction applied (*P* > 0.00625), we could think that there was a suggestive causal effect of neuroticism and SCZ increasing the risk of TMD. On the reverse MR analysis, we found no significant evidence of causal effects of TMD on these psychiatric traits. Except for heterogeneity in the causal analysis for SCZ on TMD, no heterogeneity and horizontal pleiotropy were detected in the other analyses.

**Conclusions:**

Our two-sample MR study has provided further evidence of PD and MDD being related to a higher risk of TMD.

**Clinical relevance:**

These findings highlight the importance of closely monitoring mental traits during future TMD treatments to prevent an increased risk of TMD.

**Supplementary Information:**

The online version contains supplementary material available at 10.1007/s00784-023-05339-x.

## Introduction

Temporomandibular disorders (TMD) are one of the most common chronic illnesses characterized by musculoskeletal pain that involves masticatory muscles, temporomandibular joints (TMJ), and other orofacial anatomical structures [1]. Also, it is considered to be the second-most common skeletal-muscular issue. According to a recent study that examined the prevalence of TMD in the general population, TMD affected about 31% of adults and the elderly and 11% of children and adolescents [2]. The etiopathogenesis of TMD remains unclear. In general, TMD is considered to be a disease caused by a variety of pathogenic factors, including psychological, physiological, anatomical structural, trauma, and genetic conditions [3]. These factors initiate and exacerbate the development of TMD, which causes the emergence of TMD signs and symptoms [4]. The main clinical symptoms of TMD are idiopathic and episodic musculoskeletal pain, a temporomandibular joint (TMJ) murmur (such as clicking, crepitating, and cracking), abnormal jaw movement, and associated dysfunction [5]. The presence of these symptoms, especially pain, may affect and lower the quality of life of patients, interfering with their emotional and social lives [6, 7].

In recent years, there has been a global increase in psychiatric disorders, including depression and anxiety, which pose a major burden on public health [8, 9]. Depression is the most common mental disorder and is projected to become the leading global disease burden by 2030, according to the World Health Organization [10]. A comprehensive review of 87 papers from 44 countries reported a worldwide prevalence of 7.30% for anxiety disorders [11]. Except for the high prevalence, mental illness also increases susceptibility to other diseases, thereby placing a greater burden on society. Recent studies have indicated a genetic link between major depressive disorder (MDD) and coronavirus disease 2019 [12], as well as between attention deficit hyperactivity disorder (ADHD) and diabetes [13]. Previous research has identified psychiatric disorders as potential risk factors for TMD [14, 15], with multiple observational studies demonstrating a strong association between TMD and mental symptoms like depression and anxiety [16]. Notably, over half of TMD patients exhibit symptoms of depression ranging from mild to severe [17]. Moreover, the severity of psychiatric disorders in TMD patients has been linked to treatment outcomes. Some researchers argue that psychiatric factors may impede the response of TMD patients to conservative treatment and increase their likelihood of developing chronic TMD, which can lead to disability and other adverse consequences [18–20]. Consequently, it is crucial to thoroughly investigate various types of psychiatric factors when evaluating TMD patients in order to make informed clinical decisions and initiate appropriate management strategies.

The introduction of the large-scale available GWAS database and Mendelian randomization (MR) has made it possible to infer a causal relationship between complex traits and diseases [21, 22]. MR is a method that uses genetic variants that are robustly associated with exposures as instrumental variables (IVs) to investigate the causal effects on outcomes [23]. As a novel epidemiological method, MR may be considered conceptually as natural randomized controlled trials (RCTs) because genotypes are randomly distributed from parent to offspring. Compared with observational studies, MR uses genetic variants as IVs to effectively avoid the influence of confounding factors, such as underlying bias, measurement error, and reverse causality. These confounding factors are all inherent limitations of observational studies which are difficult to control in observational studies [22]. Thus, the present study aimed to investigate the possible causation and their direction between TMD and eight psychiatric traits, including anxiety disorder (AD), panic disorder (PD), MDD, neuroticism, ADHD, autism spectrum disorder (ASD), bipolar disorder (BIP), and schizophrenia (SCZ) [24–31].

## Materials and methods

**Fig. 1:**
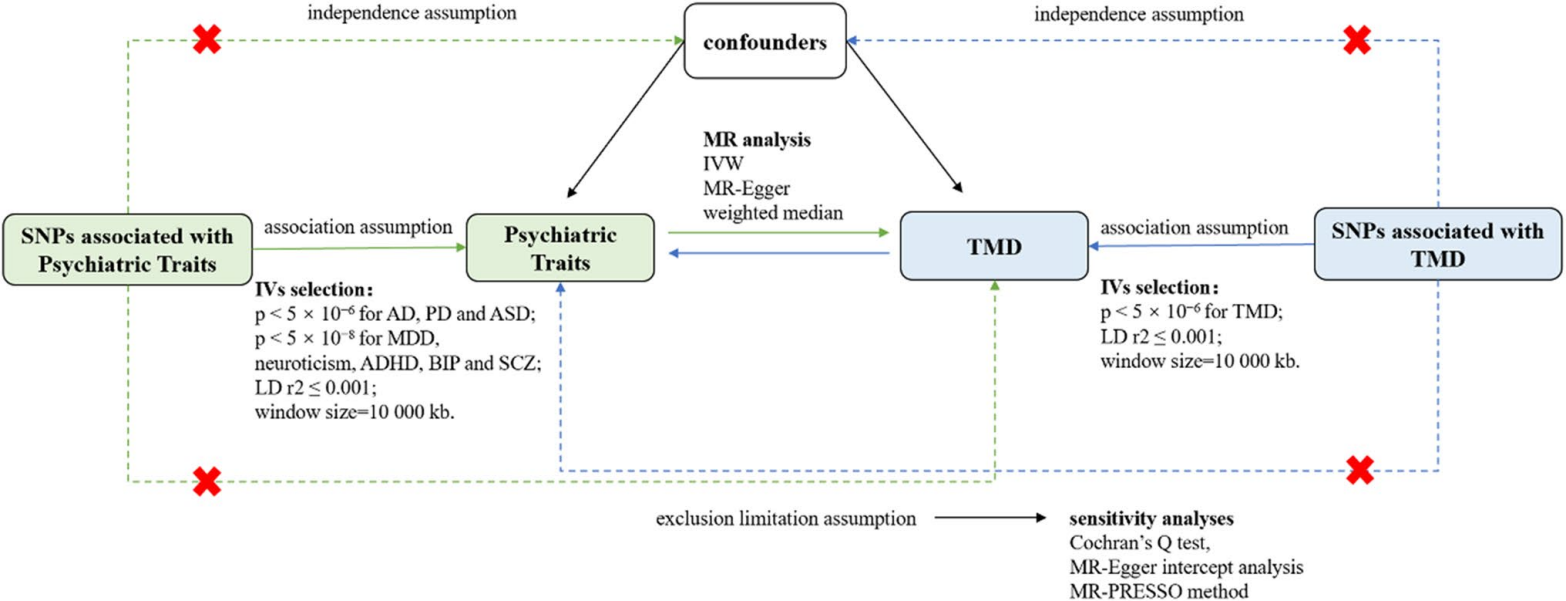
Schematic diagram of the assumption of two-sample Mendelian randomization. MR, Mendelian randomization; IVW, inverse-variance weighted; IV, instrumental variables; SNP, single nucleotide polymorphism; TMD, temporomandibular disorders; AD, anxiety disorder; PD, panic disorder; MDD, major depressive disorder; ADHD, attention deficit hyperactivity disorder; ASD, autism spectrum disorder; BIP, bipolar disorder; SCZ, schizophrenia

We employed a two-sample MR analysis to estimate the causal effects of the exposure on the outcome. To clarify the direction of causation, we performed two sets of analyses (Fig. 1): one consisting of analyses of a causal relationship between psychiatric traits on the risk of TMD and one consisting of analyses performed in the opposite direction. Since this study made use of publicly available data, informed permission from study participants as well as ethical approval were not required.

### Data source

We gathered statistics for eight psychiatric traits from the largest publicly available summary-level data derived from the GWAS meta-analysis, including the Psychiatric Genomics Consortium (PGC) and UK Biobank (UKBB) (Table 1). GWAS summary statistics of TMD were obtained from the FinnGen consortium R7 release data (http://www.finngen.fi/en). The case of TMD was defined by K07.6 in the revised International Classification of Disease (ICD-10) code. The concept set expression of TMD included the following diagnoses: “snapping jaw,” “cracking jaw joint,” “habitual dislocation of the jaw joint,” “pain in the jaw joint,” “stiffness in the jaw joint,” “temporomandibular joint-pain-dysfunction syndrome,” “temporomandibular joint disorders,” “Other disorders of the jaw joint,” and “dislocation of the jaw joint.” The study included 4273 cases and 177,661 controls for TMD. To avoid bias, there was no sample overlap between exposure and outcome cohorts, and both the two databases were from the European population in our MR studies.

**Table 1:** Description of GWAS consortiums used for eight psychiatric traits

Trait	Population	Sample size (cases/controls)	Data source	References
AD	Europeans	5580/11,730	PGC	Otowa et al. [24]
PD	Europeans	2248/7,992	PGC	Forstner et al. [25]
MDD	Europeans	170,756/329,443	PGC, UKBB	Howard et al. [26]
Neuroticism	Europeans	380,506	UKBB	Nagel et al. [27]
ADHD	96% Europeans	19,099/34,194	PGC	ADHD Working Group of the Psychiatric Genomics Consortium (PGC) et al. [28]
ASD	Europeans	18,381/27,969	PGC	Autism Spectrum Disorder Working Group of the Psychiatric Genomics Consortium et al. [29]
BIP	Europeans	20,352/31,358	PGC	eQTLGen Consortium, BIOS Consortium et al. [30]
SCZ	80% Europeans	76,755/243,649	PGC	Trubetskoy et al. [31]

*AD* anxiety disorder, *PD* panic disorder, *MDD* major depressive disorder, *ADHD* attention deficit hyperactivity disorder, *ASD* autism spectrum disorder, *BIP* bipolar disorder, *SCZ* schizophrenia, *UKBB* UK Biobank, *PGC* Psychiatric Genomics Consortium

### Genetic instrumental variable selection criteria

In principle, the selected IVs must follow the three model assumptions to satisfy the validity of MR analysis: (1) the association assumption: solidly related to the exposure, (2) the exclusion limitation assumption: influences the outcome only through exposure and not via other biological pathways, (3) the independence assumption: independent of any potential confounders [22, 32]. When three strict assumptions are met, single nucleotide polymorphisms (SNPs), which are independent genetic predictors, are considered IVs [33]. To screen the valid instrumental SNPs, we used the following settings in the R package TwoSampleMR. In order to increase the statistical effect, we chose two different *P*-value thresholds. For SNPs associated with AD, PD, ASD, and TMD, we applied a relatively lenient *P*-value threshold (*P* < 5 × 10^−6^), and the SNPs associated with the remaining exposure variable still were selected under the genome-wide significant *P* threshold < 5 × 10^−8^. Independent SNPs were selected by using standard clumping parameters (LD *r*2 ≤ 0.001; clumping window, 10 000 kb). We also calculated the *F* statistic for each IV by the following equation: *F* = *R*^2^ × (*N*−*k*−1)/*k* × (1 − *R*^2^) to assess the strength of the instruments and avoid weak instrument bias [34]. It indicated no significant weak instrumental bias if the corresponding *F* statistic was > 10 [35].

### Mendelian randomization analysis

The analyses were carried out using the R package “TwoSampleMR” to determine the correlation between the exposure and outcome. In this study, we employed three different methods: the inverse variance weighted (IVW) method, weighted median, and MR-Egger regression. IVW is the primary method in our MR analysis which is also the method that magnetorheological studies employ the most frequently. Under the absence of pleiotropy, IVW could accurately assess each valid IV and provide reliable causal estimates by combining the Wald ratio [36]. If the corresponding *P*-value < 0.05, it was considered there was causation between exposure and outcome. Furthermore, we also performed three sensitivity analyses based on Cochran’s *Q* test, MR-Egger intercept analysis, and MR-PRESSO method to illustrate the reliability of the analysis, and *P*-value > 0.05 reflected no heterogeneity or pleiotropic effect [37]. Heterogeneity was tested using Cochran’s *Q* test in the IVW method and MR-Egger regression [38]. The MR-Egger regression can identify and correct pleiotropy [39]. Moreover, we used the MR-PRESSO method, which could detect the existence of horizontal pleiotropy based on a global test, and if detected, it provides estimations obtained from this analysis that are corrected for horizontal pleiotropy via removing possible outliers [40]. To assess the potential impact of outliers, we employed the leave-one-out test. In our study, causal associations were considered statistically significant when the Bonferroni corrected *P*-value was below 0.00625 (*P* < 0.05/8). The opposite of the analysis’s direction holds as well. Furthermore, we also considered that there was regarded as suggestive evidence of causality when the *P*-value was between 0.00625 and 0.05 and further confirmation would be required.

## Results

The detailed information for the selected valid SNPs is shown in Supplementary Tables S3—S11. The *F* statistics for each IV we calculated in our study were all more than 10 (Supplementary Table S1), indicating that there were free of weak instrumental bias.

### Causal effects of psychiatric traits on TMD

**Fig. 2:**
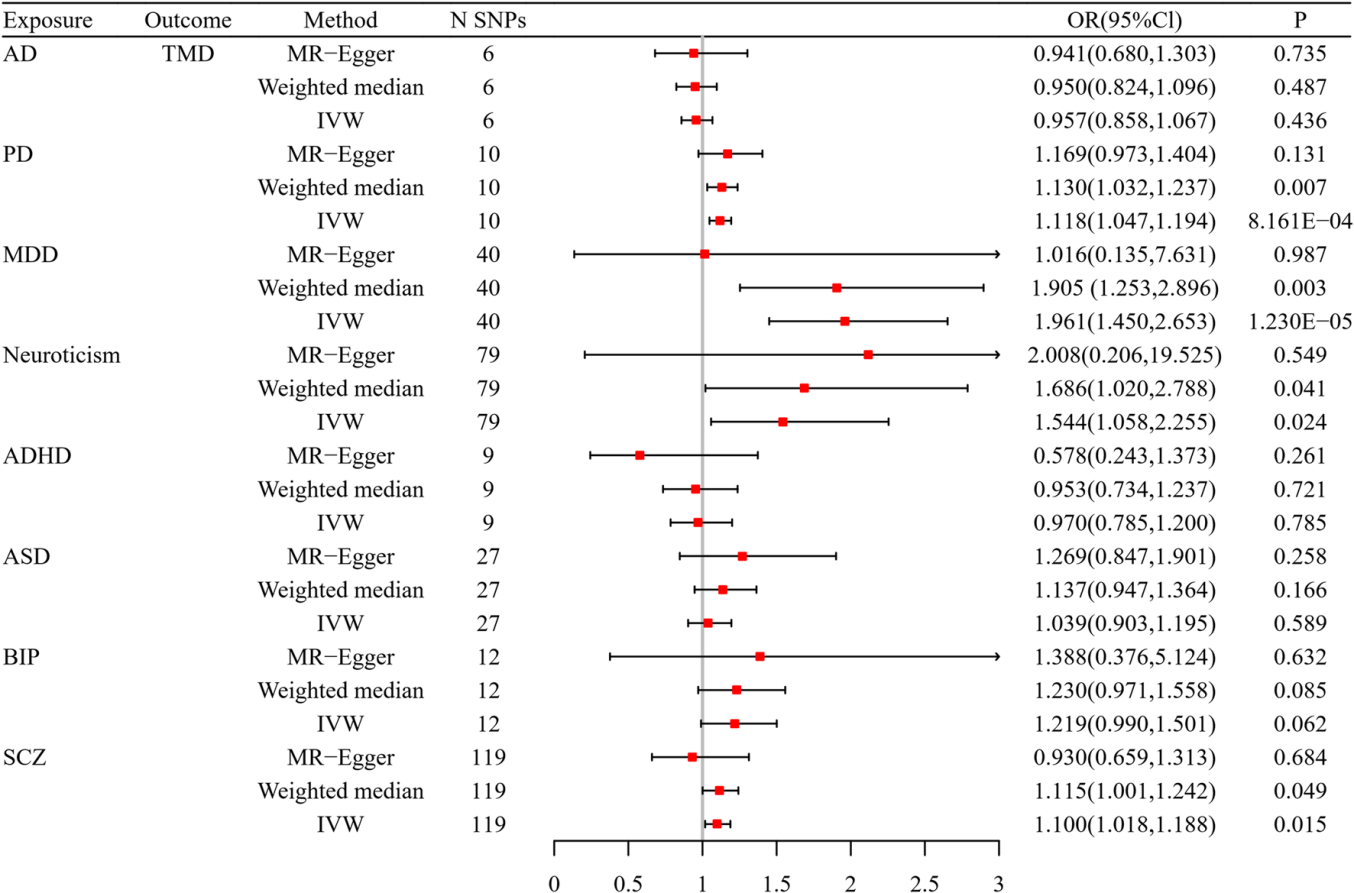
Two-sample Mendelian randomization analyses showing the effect estimates of eight psychiatric traits on TMD. IVW, inverse-variance weighted; MR, Mendelian randomization; N SNPs, number of the SNPs used in MR analysis; OR, odds ratio; CI, confidence interval; TMD, temporomandibular disorders; AD, anxiety disorder; PD, panic disorder; MDD, major depressive disorder; ADHD, attention deficit hyperactivity disorder; ASD, autism spectrum disorder; BIP, bipolar disorder; SCZ, schizophrenia

**Fig. 3:**
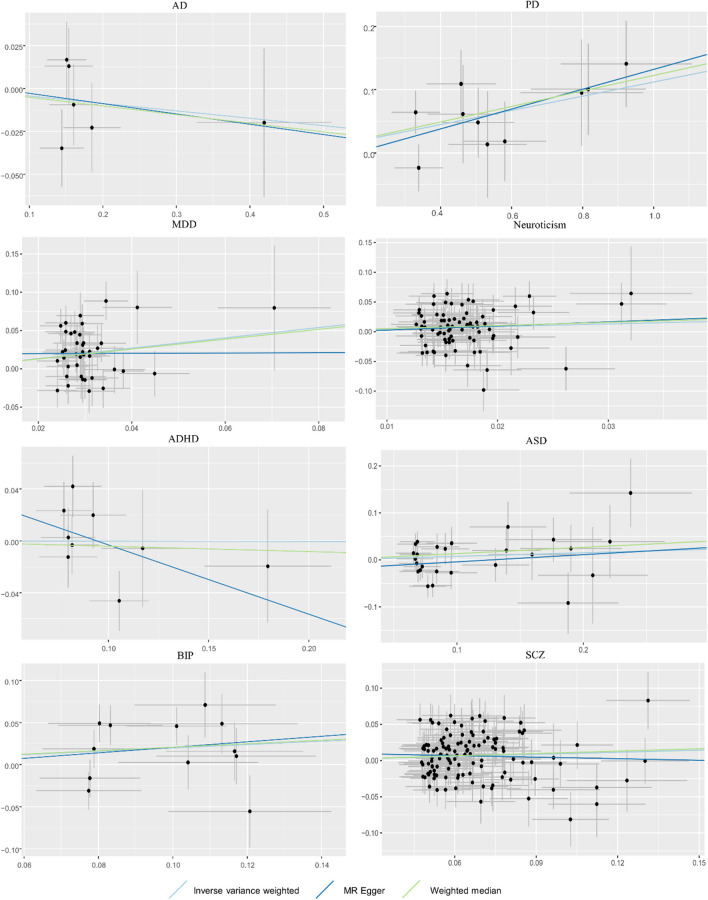
Scatter plots for the MR effect of eight psychiatric traits on risk of TMD. MR, Mendelian randomization; TMD, temporomandibular disorders; AD, anxiety disorder; PD, panic disorder; MDD, major depressive disorder; ADHD, attention deficit hyperactivity disorder; ASD, autism spectrum disorder; BIP, bipolar disorder; SCZ, schizophrenia

Figure 2 shows the MR estimates of the three methods to evaluate the causal impact of eight psychiatric traits on TMD. There were three and four outliers in the MR-PRESSO analysis of neuroticism and SCZ, respectively. And we removed these outlier SNPs and reassessed the MR analysis. Overall, we found that PD and MDD had risk effects on TMD (*P* < 0.00625). The corresponding result from the main IVW method were OR = 1.118 (95% CI: 1.047–1.194, *P* = 8.161 × 10^−4^) and OR = 1.961(95% CI: 1.450–2.653, *P* = 1.230 × 10^−5^)for PD and MDD, respectively. Despite not surpassing the strict Bonferroni correction applied (*P* > 0.00625), we could think that there was a suggestive causal effect of the neuroticism and SCZ increasing the risk of TMD (OR = 1.544, 95% CI: 1.058–2.255, *P* = 0.024; OR = 1.100, 95% CI: 1.018–1.188, *P* = 0.015). Moreover, since some mental disorders have been causally implicated in pain, we examined all the instrumental variables of PD, MDD, neuroticism, and SCZ in the Phenoscanner GWAS database (http://www.phenoscanner.medschl.cam.ac.uk/) to rule out confounders (pain). And then, we found that the association of the above psychiatric traits with TMD still remained significant after manually filtering the related SNPs from the MR analyses. Additionally, there was no association between the remaining four psychiatric traits (AD, ADHD, ASD, BIP) and TMD. Except for heterogeneity in the causal analysis for SCZ on TMD, there was no discernible heterogeneity and horizontal pleiotropy based on *Q*-tests, MR-Egger intercepts, and MR-PRESSO to identify relationships between the remaining seven psychiatric characteristics and TMD risk. Therefore, we used a random-effects IVW model when heterogeneity existed in the MR analysis of SCZ on TMD. The sensitivity analysis for the results is presented in Table 2. Scatter plots across three methods are presented in Fig. 3. The slopes of various lines show the causal relationship of various methods. The leave-one-out results of the effect of eight psychiatric traits on TMD are shown in Supplementary Figure S1).

**Table 2:** Sensitivity analyses

Exposure	Outcome	Heterogeneity test		Horizontal pleiotropy test	
		MR-Egger (*P*)	IVW (*P*)	Egger intercept (*P*)	MRPRESSO global test (*P*)
AD	TMD	0.436	0.579	0.919	0.642
PD		0.711	0.771	0.624	0.798
MDD		0.074	0.083	0.521	0.098
Neuroticism		0.304	0.328	0.771	0.318
ADHD		0.322	0.276	0.240	0.268
ASD		0.150	0.144	0.311	0.137
BIP		0.063	0.091	0.847	0.106
SCZ		0.042	0.041	0.331	0.052

*MR* Mendelian randomization, *IVW* inverse-variance weighted, *TMD* temporomandibular disorders, *AD* anxiety disorder, *PD* panic disorder, *MDD* major depressive disorder, *ADHD* attention deficit hyperactivity disorder, *ASD* autism spectrum disorder, *BIP* bipolar disorder, *SCZ* schizophrenia

### Causal effects of TMD on psychiatric traits

Reverse directional MR analysis revealed no significant causal relationship which was performed with TMD as exposure and psychiatric traits as outcomes (Supplementary Figure S2). The results of the MR-PRESSO analysis along with the results of the MR-Egger intercept analysis show that there is no horizontal pleiotropy in this analysis (the *P* values of the two tests were both greater than 0.05). And no heterogeneity was detected by the *Q*-tests (Supplementary Table S2).

## Discussion

We applied two-sample MR to investigate the causal relationship between TMD and mental disorders in this study. The results showed that PD and MDD have risk effects on TMD. Neuroticism and SCZ had a suggestive causal effect on TMD, whereas no significant evidence for causal effects was found in which TMD is relevant to these psychiatric traits in the reverse MR analyses. These findings highlight the important and complex relationship between mental disorders and TMD.

Psychiatric and neurodevelopmental disorders such as depression and anxiety stand out as comorbidities frequently associated with TMD. Numerous studies have indicated that psychiatric factors such as depression, anxiety, and SCZ are associated with an increased risk of TMD [41–44]. Through bi-directional MR studies, we have further confirmed the genetic correlation between these disorders. It has been observed that most TMD patients exhibit hyperactivity in the hypothalamic-pituitary-adrenal (HPA) axis. Mental disorders, acting as stressors, have the potential to upregulate the HPA axis in TMD patients, leading to the expression and release of corticotropin-releasing hormone (CRH), adrenocorticotropic hormone (ACTH), and cortisol [45]. This chronic activation may result in recurrent muscular hyperactivity, gradually causing damage to the joint and eventually manifesting as TMD symptoms [46]. Additionally, mental disorders can also alter brain structures such as the hippocampus and hypothalamus, interfering with the central modulation of pain responses [47]. Consequently, this can lead to an increased perception of pain, further exacerbating and promoting pain symptoms in individuals with TMD.

Multiple meta-analyses have established that inflammation caused by mental disorders, as well as the release of inflammatory cytokines, plays a crucial role in the pathophysiology of TMD. The level of inflammation in the synovial fluid of TMD patients and the blood of patients with MDD was found to be higher compared to healthy controls [48–51]. This increased cytokine activity and elevated inflammatory response can negatively affect the biomechanical properties of the disc, leading to the development and progression of TMD [52, 53]. Currently, there are several cytokines that may be involved in the pathogenesis of TMD and their relationship with pain [54]. Further analysis and exploration of these inflammatory cytokines can provide a clearer understanding of the impact of mental disorders in patients with TMD. Additionally, inhibiting these receptors may have the potential to alleviate symptoms and improve or even reverse pain conditions in patients with TMD.

In some clinical studies, other psychiatric disorders such as ADHD, ASD, and BIP have been closely associated with the prevalence of TMD. However, this MR analysis found no causal association between these three psychiatric traits and TMD. It is well known that TMD symptoms, especially pain, have also been suggested as causes or enhancers in the development of depression and psychiatric illnesses [3]. This creates a cycle where TMD and psychiatric disorders exacerbate each other’s risk. However, our study did not find evidence supporting a causal effect of TMD on these psychiatric disorders in the context of genetic variation. Therefore, it is possible that psychiatric disorders are primarily triggered by psychological stress associated with TMD rather than by genetic susceptibility to TMD.

The presence of significant genetic correlations between TMD and psychiatric disorders suggests that they cannot be considered entirely independent disease entities. TMD is significantly associated with changes in the central nervous system, suggesting a common pathophysiologic basis involving psychosocial and neuroendocrine mechanisms. Therefore, there is a great challenge to diagnosing and treating TMD patients with psychiatric disorders. In general, oral professionals generally prioritize and value attention to structural damages and symptoms of TMJ region and may neglect the mental and emotional states of these people. In order to properly assess the inducements and symptoms of TMD patients, comprehensive clinical evaluations of the mental state of patients are required. In addition, a randomized controlled trial demonstrates that TMD patients classified based on psychosocial and behavioral factors will demonstrate a differential response to the same standardized treatment [55]. The peculiarities of the psychiatric disorders may limit and influent some management of TMD, thus requiring more cooperation from the subject. In the future, a multidisciplinary collaboration between mental health professionals and oral professional teams is required to develop clinical guidelines and personalized treatment protocols for TMD patients with psychiatric disorders, in order to address the complexity of their needs and situations.

This is the first MR study to comprehensively evaluate the relationships between eight psychiatric traits and TMD. There were significant advantages to our bi-directional MR analysis. First, we selected genetic variations that were randomly assigned as IVs, to avoid the potential effects due to conventional confounders as well as reverse causation common in observational studies. Second, to prevent bias and ensure the robustness of the instruments in the MR analysis, no overlapping samples between exposures and outcomes were used [56]. Thirdly, we could more clearly determine the causal relationship and causal direction between two features by using a bi-directional MR. Moreover, our findings were reached after comprehensive studies by three MR methods, including IVW, weighted median, and MR-Egger, and several sensitivity tests demonstrated the robustness of MR analysis.

Our study had several limitations as well. First off, the sample size of TMD is relatively small, which makes the loci studied relatively limited. So, we might miss weak associations between the reverse causal association of TMD with psychiatric traits. Second, because diverse environmental factors may have significant effects on psychiatric characteristics and TMD, the findings of this study may not be totally generalized to persons of non-European heritage. Finally, women are more likely than males to experience TMD symptoms, which may vary for both psychological and physical symptoms of TMD patients [57]. Due to the GWAS summary statistics of TMD not being stratified by sex, we were unable to conduct analysis by sex stratification to confirm the sex-specific effects on causal effects between psychiatric characteristics and TMD.

## Conclusion

In conclusion, our two-sample MR study has provided further evidence of PD and MDD being related to a higher risk of TMD. However, additional studies are required to confirm the positive associations of neuroticism and SCZ with TMD. These findings highlight the importance of closely monitoring mental traits during future TMD treatments to prevent an increased risk of TMD.

### Supplementary information

ESM 1 (DOCX 1331 kb)
