# Supplementary material for: Causal relationship between psychiatric traits and temporomandibular disorders: a bidirectional two-sample Mendelian randomization study
Source: Clin Oral Investig. 2023 Nov 1;27(12):7513–21. doi: 10.1007/s00784-023-05339-x (PMC10713754; doi:10.1007/s00784-023-05339-x)
Supplement: Supplementary file 1 — (DOCX 1331 kb) [file 784_2023_5339_MOESM1_ESM.docx]

**The title: Causal Relationship Between Psychiatric Traits and Temporomandibular Disorders: A Bidirectional Two-Sample Mendelian Randomization Study**

**The journal: Clinical Oral Investigations**

**The authors: Yulin Xiang,** **Jukun Song,** **Jiaxin Sun,** **Ying Liang, Zhijun Zheng**

*** Correspondence:**

Zhijun Zheng: Department of Endodontics, Guiyang Stomatological Hospital; [zhijun.zheng@163.com](mailto:zhijun.zheng@163.com)

# Supplementary Tables

**Table S1.** F statistic between genetic instruments and exposure.

| **Exposure** | **Outcome** | **F-statistic (median)** | **F-statistic (minimum)** | **F-statistic (maximum)** |
| --- | --- | --- | --- | --- |
| AD | TMD | 223.502 | 176.043 | 390.741 |
| PD | TMD | 255.037 | 186.768 | 340.538 |
| MDD | TMD | 180.678 | 137.974 | 363.467 |
| Neuroticism | TMD | 417.256 | 305.741 | 1280.823 |
| ADHD | TMD | 172.684 | 150.871 | 243.884 |
| ASD | TMD | 111.454 | 61.461 | 153.384 |
| BIP | TMD | 160.272 | 138.009 | 265.102 |
| SCZ | TMD | 538.423 | 346.461 | 1874.072 |
| TMD | AD | 976.696 | 829.687 | 1178.322 |
| TMD | PD | 592.908 | 473.904 | 763.083 |
| TMD | MDD | 1024.866 | 796.899 | 1653.636 |
| TMD | Neuroticism | 572.520 | 348.332 | 763.083 |
| TMD | ADHD | 586.641 | 473.904 | 763.083 |
| TMD | ASD | 592.908 | 473.904 | 763.083 |
| TMD | BIP | 573.736 | 348.332 | 763.083 |
| TMD | SCZ | 1044.167 | 829.687 | 1653.636 |

TMD, temporomandibular disorders; AD, anxiety disorder; PD, panic disorder; MDD, major depressive disorder; ADHD, attention deficit hyperactivity disorder; ASD, autism spectrum disorder; BIP, bipolar disorder; SCZ, schizophrenia.

**Table S2.** Sensitivity analyses between TMD on eight psychiatric traits.

| **Exposure** | **Outcome** | **Heterogeneity test** | | **Horizontal pleiotropy test** | |  |
| --- | --- | --- | --- | --- | --- | --- |
|  |  | **MR-Egger (P)** | **IVW (P)** | **Egger**  **intercept (P)** | **MRPRESSO global Test (P)** | |
| TMD | AD | 0.730 | 0.679 | 0.293 | 0.651 | |
|  | PD | 0.349 | 0.299 | 0.572 | 0.630 | |
|  | MDD | 0.578 | 0.614 | 0.467 | 0.655 | |
|  | Neuroticism | 0.318 | 0.413 | 0.848 | 0.208 | |
|  | ADHD | 0.291 | 0.285 | 0.339 | 0.310 | |
|  | ASD | 0.106 | 0.151 | 0.215 | 0.146 | |
|  | BIP | 0.056 | 0.069 | 0.915 | 0.093 | |
|  | SCZ | 0.589 | 0.614 | 0.432 | 0.631 | |

MR, mendelian randomization; IVW, inverse-variance weighted; TMD, temporomandibular disorders; AD, anxiety disorder; PD, panic disorder; MDD, major depressive disorder; ADHD, attention deficit hyperactivity disorder; ASD, autism spectrum disorder; BIP, bipolar disorder; SCZ, schizophrenia.

**Table S3.** Characteristics of the genetic variants that were used as the instrumental variables for anxiety disorder*

| **SNP** | **Beta** | **SE** | **EAF** | **Effect allele** | **Other allele** | **P-value** |
| --- | --- | --- | --- | --- | --- | --- |
| rs1709393 | -0.1509 | 0.0267 | 0.5793 | T | C | 1.59E-08 |
| rs2146346 | 0.1441 | 0.0298 | 0.5869 | A | G | 1.33E-06 |
| rs2753188 | 0.1603 | 0.0332 | 0.7268 | A | G | 1.38E-06 |
| rs28373923 | 0.4193 | 0.0915 | 0.0675 | A | G | 4.59E-06 |
| rs58990403 | -0.1851 | 0.0391 | 0.792 | A | G | 2.20E-06 |
| rs739315 | -0.1537 | 0.0329 | 0.5688 | A | G | 2.99E-06 |

SNP, single nucleotide polymorphism; SE, standard error; EAF, effect allele frequency; Beta coefficients are in standard deviation (SD) unit per allele; effect allele is the broad depression-raising allele.

*Otowa et al., 2016[1]

**Table S4.** Characteristics of the genetic variants that were used as the instrumental variables for panic disorder*

| **SNP** | **Beta** | **SE** | **EAF** | **Effect allele** | **Other allele** | **P-value** |
| --- | --- | --- | --- | --- | --- | --- |
| rs112586150 | 0.457696962 | 0.0981 | 0.044 | A | G | 3.08E-06 |
| rs142111923 | 0.923000173 | 0.1842 | 0.0172 | A | G | 5.42E-07 |
| rs144783209 | 0.531997901 | 0.1105 | 0.0384 | T | G | 1.48E-06 |
| rs150769867 | -0.797308416 | 0.1728 | 0.981 | A | G | 3.95E-06 |
| rs1912779 | 0.329800027 | 0.0682 | 0.111 | T | G | 1.33E-06 |
| rs41280169 | 0.505497454 | 0.1013 | 0.0605 | T | C | 6.03E-07 |
| rs72737735 | 0.462997896 | 0.0994 | 0.0645 | T | C | 3.19E-06 |
| rs77338525 | 0.581097677 | 0.1163 | 0.0363 | T | C | 5.84E-07 |
| rs79918695 | -0.338400094 | 0.0681 | 0.906 | T | C | 6.72E-07 |
| rs79919349 | 0.815798348 | 0.1619 | 0.0248 | A | G | 4.68E-07 |

SNP, single nucleotide polymorphism; SE, standard error; EAF, effect allele frequency; Beta coefficients are in standard deviation (SD) unit per allele; effect allele is the broad depression-raising allele.

* Forstner et al., 2021[2]

**Table S5.** Characteristics of the genetic variants that were used as the instrumental variables for major depressive disorder*

| **SNP** | **Beta** | **SE** | **EAF** | **Effect allele** | **Other allele** | **P-value** |
| --- | --- | --- | --- | --- | --- | --- |
| rs7797112 | 0.027 | 0.0049 | 0.7467 | T | C | 3.58E-08 |
| rs9364755 | -0.0283 | 0.0051 | 0.7738 | A | G | 2.87E-08 |
| rs12631196 | 0.0241 | 0.0044 | 0.4226 | A | G | 4.32E-08 |
| rs9596774 | 0.0255 | 0.0046 | 0.3261 | T | C | 2.97E-08 |
| rs76954012 | 0.0412 | 0.0074 | 0.0931 | A | T | 2.58E-08 |
| rs3099439 | -0.0241 | 0.0043 | 0.5351 | T | C | 2.09E-08 |
| rs508502 | -0.0264 | 0.0048 | 0.2992 | T | C | 3.80E-08 |
| rs7617480 | 0.029 | 0.0051 | 0.2261 | A | C | 1.30E-08 |
| rs2488389 | -0.0295 | 0.0052 | 0.2179 | A | G | 1.40E-08 |
| rs843812 | 0.0248 | 0.0044 | 0.4117 | A | G | 1.74E-08 |
| rs28541419 | 0.0292 | 0.0052 | 0.7692 | C | G | 1.96E-08 |
| rs198457 | -0.0315 | 0.0056 | 0.1886 | T | C | 1.86E-08 |
| rs59082935 | 0.0363 | 0.0066 | 0.1342 | T | C | 3.80E-08 |
| rs4141983 | 0.0264 | 0.0046 | 0.674 | T | C | 9.52E-09 |
| rs11612312 | -0.0309 | 0.0054 | 0.7965 | T | C | 1.05E-08 |
| rs6656912 | -0.0252 | 0.0043 | 0.4273 | T | C | 4.62E-09 |
| rs2214123 | 0.0261 | 0.0045 | 0.3534 | A | G | 6.63E-09 |
| rs17737641 | -0.0705 | 0.012 | 0.9673 | A | G | 4.23E-09 |
| rs62535714 | 0.0339 | 0.0058 | 0.1639 | A | G | 5.07E-09 |
| rs56327309 | -0.0259 | 0.0044 | 0.6198 | C | G | 3.95E-09 |
| rs12040241 | 0.0259 | 0.0044 | 0.6194 | T | C | 3.95E-09 |
| rs2418449 | 0.0281 | 0.0048 | 0.719 | T | C | 4.79E-09 |
| rs12919291 | 0.0327 | 0.0055 | 0.1884 | C | G | 2.76E-09 |
| rs7152906 | -0.0258 | 0.0043 | 0.4804 | T | C | 1.97E-09 |
| rs354155 | -0.0449 | 0.0075 | 0.0923 | C | G | 2.14E-09 |
| rs66511648 | -0.0297 | 0.0048 | 0.716 | T | C | 6.11E-10 |
| rs9529314 | -0.0335 | 0.0053 | 0.2028 | A | G | 2.60E-10 |
| rs4938021 | 0.0278 | 0.0044 | 0.622 | T | C | 2.65E-10 |
| rs9074 | 0.031 | 0.0049 | 0.2597 | A | G | 2.51E-10 |
| rs12967855 | 0.0294 | 0.0046 | 0.3303 | A | G | 1.64E-10 |
| rs754287 | -0.0289 | 0.0045 | 0.3664 | A | T | 1.34E-10 |
| rs7551758 | -0.0283 | 0.0043 | 0.4671 | T | G | 4.66E-11 |
| rs1021363 | 0.03 | 0.0045 | 0.3566 | A | G | 2.62E-11 |
| rs7721129 | 0.029 | 0.0043 | 0.5343 | A | G | 1.54E-11 |
| rs1931388 | 0.0295 | 0.0044 | 0.5958 | A | G | 2.02E-11 |
| rs10501696 | 0.0295 | 0.0044 | 0.5052 | A | G | 2.02E-11 |
| rs1950829 | 0.0297 | 0.0043 | 0.4827 | A | G | 4.95E-12 |
| rs3807865 | 0.031 | 0.0044 | 0.4105 | A | G | 1.85E-12 |
| rs12967143 | -0.0345 | 0.0047 | 0.7012 | C | G | 2.13E-13 |
| rs2568958 | 0.0382 | 0.0044 | 0.6042 | A | G | 3.89E-18 |

SNP, single nucleotide polymorphism; SE, standard error; EAF, effect allele frequency; Beta coefficients are in standard deviation (SD) unit per allele; effect allele is the broad depression-raising allele.

*****Howard et al., 2019[3]

**Table S6.** Characteristics of the genetic variants that were used as the instrumental variables for neuroticism*

| **SNP** | **Beta** | **SE** | **EAF** | **Effect allele** | **Other allele** | **P-value** |
| --- | --- | --- | --- | --- | --- | --- |
| rs10494048 | 0.0207156 | 0.00245231 | 0.40538 | T | G | 2.98E-17 |
| rs1064213 | 0.0207325 | 0.00241093 | 0.5056 | G | A | 8.01E-18 |
| rs10761231 | -0.0220634 | 0.00254386 | 0.34475 | G | A | 4.20E-18 |
| rs10801874 | -0.0202191 | 0.00273474 | 0.26997 | T | C | 1.43E-13 |
| rs10877022 | -0.0193128 | 0.00263354 | 0.30314 | G | A | 2.24E-13 |
| rs10893052 | -0.0210625 | 0.00255296 | 0.66232 | T | A | 1.58E-16 |
| rs10896647 | 0.0190592 | 0.00257206 | 0.32519 | T | C | 1.26E-13 |
| rs10930502 | 0.0196384 | 0.0026611 | 0.3047 | G | A | 1.59E-13 |
| rs11662842 | 0.0233549 | 0.00255165 | 0.35404 | G | C | 5.54E-20 |
| rs11663217 | 0.0205317 | 0.00244615 | 0.44465 | G | A | 4.72E-17 |
| rs11729015 | 0.0178912 | 0.00245987 | 0.5102 | C | A | 3.51E-13 |
| rs118166957 | 0.0302886 | 0.00343691 | 0.15473 | T | C | 1.22E-18 |
| rs11865749 | -0.0211571 | 0.00255333 | 0.39096 | G | C | 1.17E-16 |
| rs12272545 | -0.0252392 | 0.00287538 | 0.23487 | G | A | 1.67E-18 |
| rs12561863 | 0.0195956 | 0.00243337 | 0.43509 | T | A | 8.09E-16 |
| rs12684080 | -0.0486159 | 0.00538455 | 0.05711 | T | C | 1.74E-19 |
| rs12713388 | 0.0189959 | 0.00245539 | 0.58081 | G | A | 1.02E-14 |
| rs12821431 | -0.0284579 | 0.00301217 | 0.20654 | T | C | 3.47E-21 |
| rs12927162 | 0.0276071 | 0.00272465 | 0.2563 | G | A | 3.97E-24 |
| rs12928387 | -0.0209054 | 0.0026897 | 0.71929 | G | C | 7.70E-15 |
| rs13037010 | 0.0315806 | 0.00325809 | 0.82077 | G | A | 3.23E-22 |
| rs13095140 | 0.0260911 | 0.00291527 | 0.77846 | T | C | 3.56E-19 |
| rs1349490 | 0.0183948 | 0.00244091 | 0.47217 | T | C | 4.84E-14 |
| rs1358393 | -0.0276327 | 0.00242006 | 0.49041 | T | G | 3.39E-30 |
| rs1411751 | -0.0233746 | 0.00296479 | 0.79331 | G | A | 3.17E-15 |
| rs1533305 | -0.0213692 | 0.00269843 | 0.72387 | G | A | 2.39E-15 |
| rs1789695 | 0.0193244 | 0.00260012 | 0.31528 | C | A | 1.07E-13 |
| rs1823125 | -0.0345978 | 0.00288865 | 0.22969 | G | A | 4.68E-33 |
| rs1846644 | 0.0223759 | 0.00247342 | 0.60091 | T | C | 1.48E-19 |
| rs1935884 | -0.0305638 | 0.00253886 | 0.66133 | T | C | 2.23E-33 |
| rs1939252 | 0.0181365 | 0.00242351 | 0.47028 | G | C | 7.23E-14 |
| rs1984839 | -0.0209915 | 0.00258308 | 0.67866 | G | C | 4.42E-16 |
| rs2242090 | 0.0200417 | 0.0024534 | 0.53343 | G | C | 3.11E-16 |
| rs2344121 | -0.0193317 | 0.00246298 | 0.46302 | G | A | 4.20E-15 |
| rs2431108 | -0.0245468 | 0.00257516 | 0.66656 | T | C | 1.54E-21 |
| rs2503185 | -0.0185079 | 0.00242518 | 0.47416 | G | A | 2.32E-14 |
| rs2568960 | 0.0318518 | 0.00251197 | 0.63437 | G | A | 7.63E-37 |
| rs2664299 | -0.0185188 | 0.00246767 | 0.59096 | T | C | 6.16E-14 |
| rs2718777 | 0.0193344 | 0.0025324 | 0.64013 | T | C | 2.26E-14 |
| rs293567 | 0.0276958 | 0.00261194 | 0.33886 | T | G | 2.87E-26 |
| rs34227359 | 0.0284943 | 0.00398674 | 0.88028 | T | C | 8.85E-13 |
| rs356179 | 0.0215031 | 0.00256108 | 0.66304 | G | C | 4.62E-17 |
| rs35672980 | 0.022226 | 0.0029162 | 0.77299 | T | A | 2.51E-14 |
| rs35681248 | -0.0310561 | 0.00399193 | 0.88885 | T | C | 7.27E-15 |
| rs3732730 | 0.0408674 | 0.00426015 | 0.90963 | G | A | 8.56E-22 |
| rs3814883 | -0.0181452 | 0.00249973 | 0.47612 | T | C | 3.90E-13 |
| rs3824345 | 0.0181566 | 0.00253978 | 0.36698 | T | A | 8.75E-13 |
| rs4128928 | -0.0222925 | 0.00254657 | 0.34052 | G | C | 2.06E-18 |
| rs4297935 | -0.021973 | 0.00269354 | 0.29155 | T | G | 3.42E-16 |
| rs4304930 | -0.0372838 | 0.00243947 | 0.53685 | C | A | 9.84E-53 |
| rs4383496 | -0.0223826 | 0.00241387 | 0.47936 | T | C | 1.82E-20 |
| rs4595203 | 0.0300002 | 0.00277226 | 0.25171 | G | A | 2.72E-27 |
| rs463245 | -0.0178963 | 0.00250426 | 0.6048 | T | C | 8.91E-13 |
| rs4664299 | -0.0215174 | 0.00289024 | 0.23514 | T | C | 9.71E-14 |
| rs4674625 | 0.0181622 | 0.00242749 | 0.47131 | T | C | 7.33E-14 |
| rs4702 | 0.0300431 | 0.00241451 | 0.45585 | G | A | 1.53E-35 |
| rs4721061 | -0.0186423 | 0.00247802 | 0.59467 | G | C | 5.35E-14 |
| rs4784655 | -0.0210036 | 0.00256869 | 0.66913 | G | C | 2.92E-16 |
| rs4799681 | -0.0309716 | 0.00386534 | 0.89014 | T | G | 1.12E-15 |
| rs4955420 | 0.0183341 | 0.00254112 | 0.33926 | T | C | 5.39E-13 |
| rs4977893 | 0.0250671 | 0.00321148 | 0.82323 | T | C | 5.93E-15 |
| rs4981170 | 0.0247434 | 0.00315816 | 0.809 | G | A | 4.70E-15 |
| rs55728265 | 0.026287 | 0.0029843 | 0.20823 | T | C | 1.27E-18 |
| rs56182580 | -0.0220817 | 0.00273526 | 0.73055 | T | C | 6.86E-16 |
| rs56410336 | -0.0192406 | 0.00252443 | 0.6301 | T | C | 2.50E-14 |
| rs6982265 | -0.0181792 | 0.00244675 | 0.43226 | T | C | 1.09E-13 |
| rs7044885 | 0.0195444 | 0.00246251 | 0.55912 | G | C | 2.07E-15 |
| rs7072776 | -0.0195278 | 0.00263891 | 0.70505 | G | A | 1.36E-13 |
| rs7162423 | -0.0219414 | 0.00242359 | 0.46076 | T | C | 1.39E-19 |
| rs7184998 | 0.0227676 | 0.00243795 | 0.51257 | T | C | 9.74E-21 |
| rs72807818 | 0.0304034 | 0.00363581 | 0.87187 | G | A | 6.16E-17 |
| rs72808262 | 0.0218641 | 0.00290509 | 0.77173 | T | C | 5.23E-14 |
| rs73034109 | -0.0207443 | 0.00290418 | 0.77926 | G | A | 9.14E-13 |
| rs75218827 | 0.065094 | 0.00813836 | 0.02702 | T | C | 1.26E-15 |
| rs75742406 | 0.0210318 | 0.00284895 | 0.75217 | G | A | 1.56E-13 |
| rs77176363 | 0.0251635 | 0.0031958 | 0.81107 | G | A | 3.44E-15 |
| rs78989434 | -0.0242566 | 0.00296075 | 0.77194 | T | G | 2.55E-16 |
| rs7915425 | -0.0222937 | 0.00308395 | 0.19186 | T | C | 4.87E-13 |
| rs7940070 | -0.0196551 | 0.00266045 | 0.29606 | T | A | 1.49E-13 |
| rs79693059 | 0.0346802 | 0.00440611 | 0.08432 | G | C | 3.52E-15 |
| rs8076183 | -0.0200887 | 0.00245598 | 0.45403 | T | C | 2.85E-16 |
| rs9317586 | -0.0213739 | 0.00243567 | 0.50878 | C | A | 1.70E-18 |
| rs9375945 | 0.0187206 | 0.00244704 | 0.42581 | T | A | 2.01E-14 |
| rs9570214 | -0.0307849 | 0.00399152 | 0.89446 | G | C | 1.23E-14 |
| rs9787523 | 0.0207972 | 0.00247432 | 0.57465 | T | C | 4.27E-17 |
| rs9889282 | 0.0207652 | 0.00249852 | 0.38876 | C | A | 9.49E-17 |
| rs9938120 | 0.0287012 | 0.00339781 | 0.14709 | T | C | 2.99E-17 |

SNP, single nucleotide polymorphism; SE, standard error; EAF, effect allele frequency; Beta coefficients are in standard deviation (SD) unit per allele; effect allele is the broad depression-raising allele.

***** Nagel et al., 2018[4]

**Table S7.** Characteristics of the genetic variants that were used as the instrumental variables for attention deficit hyperactivity disorder*

| **SNP** | **Beta** | **SE** | **EAF** | **Effect allele** | **Other allele** | **P-value** |
| --- | --- | --- | --- | --- | --- | --- |
| rs11591402 | -0.092400417 | 0.0164 | 0.212 | A | T | 1.76E-08 |
| rs17531412 | 0.105395515 | 0.0148 | 0.712 | A | G | 1.07E-12 |
| rs212178 | -0.117095772 | 0.0205 | 0.871 | A | G | 1.12E-08 |
| rs4858241 | 0.082197308 | 0.0143 | 0.636 | T | G | 9.03E-09 |
| rs4916723 | -0.077799392 | 0.0138 | 0.552 | A | C | 1.72E-08 |
| rs704061 | -0.08190444 | 0.0135 | 0.547 | T | C | 1.30E-09 |
| rs74760947 | -0.179605249 | 0.0317 | 0.949 | A | G | 1.46E-08 |
| rs8039398 | -0.079996042 | 0.0135 | 0.533 | T | C | 3.11E-09 |
| rs9969232 | 0.079901159 | 0.0145 | 0.67 | A | G | 3.58E-08 |

SNP, single nucleotide polymorphism; SE, standard error; EAF, effect allele frequency; Beta coefficients are in standard deviation (SD) unit per allele; effect allele is the broad depression-raising allele.

***** ADHD Working Group of the Psychiatric Genomics Consortium (PGC) et al., 2019[5]

**Table S8.** Characteristics of the genetic variants that were used as the instrumental variables for autism spectrum disorder*

| **SNP** | **Beta** | **SE** | **EAF** | **Effect allele** | **Other allele** | **P-value** |
| --- | --- | --- | --- | --- | --- | --- |
| rs10099100 | 0.084304383 | 0.0147 | 0.341 | C | G | 9.75E-09 |
| rs10110094 | 0.090699567 | 0.0191 | 0.1451 | A | G | 2.05E-06 |
| rs11185408 | -0.068696487 | 0.0138 | 0.5089 | A | G | 6.42E-07 |
| rs112635299 | 0.22099725 | 0.0432 | 0.9831 | T | G | 3.13E-07 |
| rs11787216 | -0.069200036 | 0.0147 | 0.667 | T | C | 2.51E-06 |
| rs144911765 | -0.190095697 | 0.0403 | 0.0268 | T | C | 2.39E-06 |
| rs1452075 | 0.080704027 | 0.0155 | 0.2843 | T | C | 1.92E-07 |
| rs149923766 | -0.237305865 | 0.0484 | 0.0119 | T | G | 9.44E-07 |
| rs16879023 | -0.095795297 | 0.0201 | 0.1521 | A | G | 1.88E-06 |
| rs2224274 | 0.070998858 | 0.0138 | 0.498 | T | C | 2.68E-07 |
| rs292441 | -0.072495427 | 0.0149 | 0.327 | A | G | 1.14E-06 |
| rs35404050 | 0.084304383 | 0.0176 | 0.7883 | T | C | 1.67E-06 |
| rs45595836 | 0.138996432 | 0.0272 | 0.9066 | T | C | 3.22E-07 |
| rs4750990 | -0.068096844 | 0.0141 | 0.3857 | T | C | 1.37E-06 |
| rs644552 | 0.159402579 | 0.0346 | 0.0626 | A | G | 4.08E-06 |
| rs6692705 | 0.065600457 | 0.0141 | 0.3907 | A | G | 3.28E-06 |
| rs740883 | -0.113695152 | 0.0238 | 0.0974 | A | T | 1.78E-06 |
| rs76397219 | -0.140297086 | 0.0303 | 0.9175 | A | G | 3.65E-06 |
| rs77691144 | -0.207405546 | 0.0435 | 0.0318 | T | C | 1.86E-06 |
| rs7783557 | 0.067004229 | 0.0146 | 0.3211 | T | C | 4.45E-06 |
| rs78058104 | 0.187897648 | 0.0397 | 0.0328 | A | G | 2.21E-06 |
| rs78653484 | -0.176295748 | 0.0385 | 0.9493 | T | C | 4.67E-06 |
| rs78827416 | 0.130501808 | 0.0266 | 0.0746 | A | G | 9.29E-07 |
| rs79940520 | -0.095399184 | 0.0207 | 0.8519 | A | G | 4.05E-06 |
| rs910805 | -0.095696254 | 0.016 | 0.7644 | A | G | 2.22E-09 |
| rs9366877 | 0.068499416 | 0.0139 | 0.5726 | A | G | 8.31E-07 |
| rs9389208 | 0.0672006 | 0.0144 | 0.6233 | T | C | 3.06E-06 |

SNP, single nucleotide polymorphism; SE, standard error; EAF, effect allele frequency; Beta coefficients are in standard deviation (SD) unit per allele; effect allele is the broad depression-raising allele.

* Autism Spectrum Disorder Working Group of the Psychiatric Genomics Consortium et al., 2019[6]

**Table 9.** Characteristics of the genetic variants that were used as the instrumental variables for bipolar disorder*

| **SNP** | **Beta** | **SE** | **EAF** | **Effect allele** | **Other allele** | **P-value** |
| --- | --- | --- | --- | --- | --- | --- |
| rs10744560 | 0.083201 | 0.014 | 0.342297 | T | C | 2.92E-09 |
| rs111444407 | 0.1166 | 0.0184 | 0.154691 | T | C | 2.40E-10 |
| rs11724116 | -0.104095 | 0.0188 | 0.155277 | T | C | 3.27E-08 |
| rs13231398 | -0.1207 | 0.0219 | 0.107064 | C | G | 3.36E-08 |
| rs17150022 | 0.113202 | 0.0204 | 0.120723 | C | T | 2.70E-08 |
| rs174592 | 0.0774 | 0.0141 | 0.372297 | G | A | 3.66E-08 |
| rs2071044 | -0.077702 | 0.0135 | 0.467916 | T | C | 9.09E-09 |
| rs329319 | -0.078802 | 0.0139 | 0.565703 | G | A | 1.54E-08 |
| rs55648125 | 0.117096 | 0.0215 | 0.108936 | G | A | 4.92E-08 |
| rs73496688 | 0.108702 | 0.019 | 0.149117 | A | T | 1.05E-08 |
| rs884301 | 0.080298 | 0.0138 | 0.381084 | T | C | 5.80E-09 |
| rs9834970 | 0.101003 | 0.0134 | 0.501233 | C | T | 5.53E-14 |

SNP, single nucleotide polymorphism; SE, standard error; EAF, effect allele frequency; Beta coefficients are in standard deviation (SD) unit per allele; effect allele is the broad depression-raising allele.

* eQTLGen Consortium, BIOS Consortium et al., 2019[7]

**Table S10.** Characteristics of the genetic variants that were used as the instrumental variables for schizophrenia*

| **SNP** | **Beta** | **SE** | **EAF** | **Effect allele** | **Other allele** | **P-value** |
| --- | --- | --- | --- | --- | --- | --- |
| rs1000237 | -0.073205303 | 0.0089 | 0.627 | T | A | 1.95E-16 |
| rs10035564 | -0.066802415 | 0.0092 | 0.65 | A | G | 3.84E-13 |
| rs10086619 | -0.072205168 | 0.0116 | 0.831 | A | G | 4.83E-10 |
| rs10108980 | -0.062801364 | 0.0106 | 0.785 | C | T | 3.13E-09 |
| rs10117 | 0.054999418 | 0.0088 | 0.613 | G | A | 4.11E-10 |
| rs10160905 | -0.087204123 | 0.0144 | 0.897 | T | G | 1.40E-09 |
| rs10861176 | -0.055502139 | 0.0098 | 0.257 | G | A | 1.48E-08 |
| rs10876446 | -0.054002223 | 0.0094 | 0.674 | G | C | 9.20E-09 |
| rs11027839 | -0.051503843 | 0.0086 | 0.488 | A | C | 2.11E-09 |
| rs11165868 | -0.075402656 | 0.0117 | 0.833 | C | T | 1.16E-10 |
| rs11223774 | 0.052497569 | 0.0094 | 0.301 | A | G | 2.34E-08 |
| rs113264400 | -0.112295619 | 0.0202 | 0.948 | T | C | 2.71E-08 |
| rs11587347 | -0.103894923 | 0.0147 | 0.895 | C | G | 1.58E-12 |
| rs11693094 | 0.054402954 | 0.0087 | 0.556 | C | T | 4.02E-10 |
| rs117178087 | 0.096400494 | 0.0177 | 0.939 | C | T | 5.14E-08 |
| rs11941714 | 0.051595749 | 0.0093 | 0.673 | G | A | 2.89E-08 |
| rs12129573 | -0.077799392 | 0.0089 | 0.616 | C | A | 2.30E-18 |
| rs12138231 | -0.066994869 | 0.0116 | 0.171 | T | A | 7.68E-09 |
| rs12285419 | -0.084904507 | 0.011 | 0.8 | C | A | 1.18E-14 |
| rs12293670 | 0.070495742 | 0.0092 | 0.678 | A | G | 1.82E-14 |
| rs12412038 | 0.131203686 | 0.0154 | 0.92 | G | A | 1.60E-17 |
| rs12489270 | -0.057904575 | 0.0089 | 0.612 | T | C | 7.71E-11 |
| rs12652777 | 0.048799688 | 0.0086 | 0.489 | T | C | 1.39E-08 |
| rs12712510 | 0.057400607 | 0.0087 | 0.488 | T | C | 4.17E-11 |
| rs12771371 | 0.052402679 | 0.0093 | 0.697 | G | A | 1.75E-08 |
| rs12877581 | -0.059601395 | 0.0099 | 0.716 | G | C | 1.74E-09 |
| rs12883788 | -0.061301101 | 0.0087 | 0.527 | C | T | 1.84E-12 |
| rs13016542 | 0.088303875 | 0.0129 | 0.876 | T | C | 7.63E-12 |
| rs13233308 | 0.048704446 | 0.0086 | 0.526 | C | T | 1.48E-08 |
| rs132582 | 0.050997251 | 0.0086 | 0.471 | C | T | 3.03E-09 |
| rs1427633 | 0.048304332 | 0.0088 | 0.419 | G | C | 4.04E-08 |
| rs1430894 | -0.053295297 | 0.0086 | 0.506 | C | T | 5.75E-10 |
| rs145071536 | -0.085100477 | 0.012 | 0.799 | T | C | 1.32E-12 |
| rs1451488 | -0.070894679 | 0.0087 | 0.432 | A | G | 3.68E-16 |
| rs149165 | 0.048199514 | 0.0087 | 0.569 | T | G | 3.02E-08 |
| rs1593304 | -0.064101283 | 0.0111 | 0.193 | A | G | 7.70E-09 |
| rs167924 | -0.050199156 | 0.009 | 0.359 | A | G | 2.44E-08 |
| rs16851048 | -0.074497279 | 0.0107 | 0.792 | T | C | 3.35E-12 |
| rs16867571 | 0.065703467 | 0.0104 | 0.778 | A | G | 2.66E-10 |
| rs17149781 | -0.085601462 | 0.0137 | 0.885 | A | G | 4.15E-10 |
| rs17194490 | -0.078199408 | 0.0116 | 0.827 | G | T | 1.57E-11 |
| rs17731 | -0.052399169 | 0.0089 | 0.62 | G | A | 3.92E-09 |
| rs1860002 | 0.083798722 | 0.0087 | 0.479 | C | T | 5.86E-22 |
| rs187557 | 0.066695568 | 0.0119 | 0.163 | C | T | 2.09E-08 |
| rs1901512 | 0.058400975 | 0.0094 | 0.318 | T | C | 5.20E-10 |
| rs1915019 | 0.057098412 | 0.0098 | 0.265 | A | G | 5.66E-09 |
| rs2053079 | -0.059898635 | 0.0101 | 0.754 | A | G | 3.02E-09 |
| rs2078266 | 0.069600687 | 0.0126 | 0.178 | A | G | 3.32E-08 |
| rs215412 | -0.057703268 | 0.0091 | 0.661 | G | A | 2.28E-10 |
| rs217339 | 0.050303305 | 0.0087 | 0.583 | C | T | 7.38E-09 |
| rs2304205 | 0.068900867 | 0.0101 | 0.753 | A | C | 8.99E-12 |
| rs2332700 | 0.075098196 | 0.0099 | 0.258 | C | G | 3.31E-14 |
| rs2333321 | 0.071203759 | 0.0105 | 0.218 | A | G | 1.19E-11 |
| rs2381411 | -0.050398958 | 0.0088 | 0.586 | T | C | 1.02E-08 |
| rs2414718 | -0.063696209 | 0.0087 | 0.4 | G | A | 2.45E-13 |
| rs2455415 | -0.049494912 | 0.0088 | 0.577 | C | T | 1.86E-08 |
| rs2456020 | 0.08159842 | 0.0102 | 0.773 | C | T | 1.25E-15 |
| rs2514218 | 0.070495742 | 0.0092 | 0.668 | C | T | 1.82E-14 |
| rs2532240 | 0.060803429 | 0.0091 | 0.61 | C | T | 2.36E-11 |
| rs2710323 | 0.078404443 | 0.0086 | 0.531 | T | C | 7.74E-20 |
| rs2815731 | 0.060003252 | 0.0091 | 0.66 | C | A | 4.29E-11 |
| rs2909457 | 0.048999666 | 0.0087 | 0.451 | G | A | 1.78E-08 |
| rs2999391 | -0.051798685 | 0.0094 | 0.299 | A | G | 3.58E-08 |
| rs308697 | 0.050103587 | 0.0087 | 0.574 | C | A | 8.46E-09 |
| rs3115366 | 0.061104507 | 0.0086 | 0.524 | G | A | 1.20E-12 |
| rs35734242 | -0.050703994 | 0.0089 | 0.562 | T | C | 1.22E-08 |
| rs3739118 | 0.057003957 | 0.0095 | 0.719 | G | A | 1.97E-09 |
| rs3770754 | 0.052896009 | 0.0091 | 0.645 | C | G | 6.15E-09 |
| rs3791710 | 0.060003252 | 0.0108 | 0.804 | T | C | 2.76E-08 |
| rs3802924 | 0.073603559 | 0.0108 | 0.805 | A | C | 9.42E-12 |
| rs3814883 | 0.067097744 | 0.0087 | 0.548 | C | T | 1.23E-14 |
| rs4129585 | 0.074996149 | 0.0087 | 0.456 | A | C | 6.68E-18 |
| rs4441417 | 0.050303305 | 0.0091 | 0.67 | C | T | 3.24E-08 |
| rs4575535 | -0.055798163 | 0.0096 | 0.281 | A | G | 6.16E-09 |
| rs4632195 | -0.047196435 | 0.0086 | 0.471 | C | T | 4.07E-08 |
| rs4636654 | 0.048304332 | 0.0089 | 0.608 | G | A | 5.72E-08 |
| rs4653164 | -0.051103839 | 0.0092 | 0.323 | C | T | 2.78E-08 |
| rs4702 | 0.084304383 | 0.0089 | 0.461 | G | A | 2.73E-21 |
| rs4766428 | -0.075003758 | 0.0089 | 0.539 | C | T | 3.53E-17 |
| rs4779050 | 0.057995286 | 0.0089 | 0.381 | T | G | 7.21E-11 |
| rs4921741 | -0.055999086 | 0.0098 | 0.729 | A | G | 1.10E-08 |
| rs498591 | -0.072495427 | 0.0121 | 0.847 | A | T | 2.08E-09 |
| rs500102 | 0.051700212 | 0.0088 | 0.413 | T | C | 4.23E-09 |
| rs505061 | -0.053495718 | 0.0086 | 0.495 | C | A | 4.96E-10 |
| rs56205728 | -0.0630037 | 0.0097 | 0.7 | G | A | 8.29E-11 |
| rs56335113 | 0.064701008 | 0.0094 | 0.316 | A | G | 5.86E-12 |
| rs57433322 | 0.083099569 | 0.0139 | 0.885 | C | G | 2.25E-09 |
| rs5751191 | -0.065595079 | 0.0086 | 0.484 | T | C | 2.40E-14 |
| rs58120505 | 0.089603016 | 0.0088 | 0.602 | T | C | 2.38E-24 |
| rs6125656 | -0.064495855 | 0.0111 | 0.809 | G | A | 6.23E-09 |
| rs61937595 | 0.130098005 | 0.0162 | 0.917 | C | T | 9.69E-16 |
| rs62183855 | 0.066096682 | 0.0111 | 0.814 | A | C | 2.61E-09 |
| rs62266065 | 0.074002967 | 0.0134 | 0.888 | C | T | 3.34E-08 |
| rs634940 | -0.066396246 | 0.0099 | 0.737 | G | T | 1.99E-11 |
| rs6482437 | -0.098903629 | 0.0142 | 0.0994 | A | C | 3.28E-12 |
| rs6520064 | -0.058498134 | 0.0106 | 0.787 | A | G | 3.42E-08 |
| rs6538539 | 0.056796126 | 0.0086 | 0.462 | G | T | 4.00E-11 |
| rs6546857 | -0.060397773 | 0.0102 | 0.757 | A | G | 3.19E-09 |
| rs6549963 | 0.048304332 | 0.0088 | 0.596 | T | C | 4.04E-08 |
| rs6673880 | -0.062301026 | 0.0091 | 0.492 | A | G | 7.58E-12 |
| rs6715366 | -0.054097222 | 0.0097 | 0.723 | G | A | 2.45E-08 |
| rs6721531 | 0.051700212 | 0.0091 | 0.666 | A | T | 1.34E-08 |
| rs6943762 | 0.105098481 | 0.0132 | 0.883 | T | C | 1.69E-15 |
| rs6974218 | 0.054895299 | 0.0089 | 0.634 | A | C | 6.92E-10 |
| rs6984242 | 0.054696497 | 0.0087 | 0.413 | G | A | 3.24E-10 |
| rs704367 | 0.061499533 | 0.0093 | 0.316 | A | C | 3.77E-11 |
| rs7112616 | 0.05220338 | 0.0086 | 0.515 | T | C | 1.28E-09 |
| rs713692 | -0.056602098 | 0.0095 | 0.299 | G | A | 2.55E-09 |
| rs7174732 | -0.058402717 | 0.0097 | 0.265 | C | A | 1.73E-09 |
| rs72802868 | 0.069199517 | 0.0096 | 0.724 | G | T | 5.67E-13 |
| rs728055 | 0.067396932 | 0.009 | 0.658 | T | A | 6.96E-14 |
| rs72943392 | -0.053495718 | 0.0096 | 0.707 | G | C | 2.51E-08 |
| rs72986630 | -0.112295619 | 0.0179 | 0.926 | C | T | 3.53E-10 |
| rs73229090 | 0.102601714 | 0.0142 | 0.897 | C | A | 4.99E-13 |
| rs73292401 | -0.06760455 | 0.0109 | 0.799 | T | A | 5.57E-10 |
| rs7464611 | 0.051700212 | 0.0087 | 0.444 | C | T | 2.81E-09 |
| rs7515363 | 0.053502852 | 0.0089 | 0.389 | C | T | 1.84E-09 |
| rs7575796 | 0.096300598 | 0.0172 | 0.917 | A | G | 2.16E-08 |
| rs7634476 | -0.057703268 | 0.0088 | 0.397 | A | G | 5.48E-11 |
| rs7647398 | 0.077497934 | 0.0109 | 0.811 | C | T | 1.16E-12 |
| rs76838079 | -0.078004786 | 0.0138 | 0.849 | C | T | 1.58E-08 |
| rs7701440 | -0.06980034 | 0.0086 | 0.497 | T | C | 4.81E-16 |
| rs778371 | -0.080602865 | 0.0095 | 0.699 | A | G | 2.17E-17 |
| rs7798283 | 0.074002967 | 0.0134 | 0.875 | T | G | 3.34E-08 |
| rs7830315 | -0.04780465 | 0.0086 | 0.478 | T | C | 2.72E-08 |
| rs7867465 | -0.065402895 | 0.0117 | 0.836 | C | T | 2.27E-08 |
| rs79445414 | -0.123400031 | 0.0222 | 0.956 | T | C | 2.72E-08 |
| rs8055219 | -0.066503116 | 0.0101 | 0.758 | G | A | 4.57E-11 |
| rs9304548 | 0.056701643 | 0.01 | 0.261 | C | A | 1.43E-08 |
| rs9318627 | 0.061198575 | 0.0088 | 0.613 | A | C | 3.54E-12 |
| rs9461916 | -0.053295297 | 0.0088 | 0.388 | T | C | 1.39E-09 |
| rs9636107 | -0.069896851 | 0.0086 | 0.506 | A | G | 4.38E-16 |
| rs9876421 | -0.06250326 | 0.0092 | 0.645 | C | T | 1.09E-11 |

SNP, single nucleotide polymorphism; SE, standard error; EAF, effect allele frequency; Beta coefficients are in standard deviation (SD) unit per allele; effect allele is the broad depression-raising allele.

*Trubetskoy et al., 2022[8]

**Table S11.** Characteristics of the genetic variants that were used as the instrumental variables for Temporomandibular disorders*

| **SNP** | **Beta** | **SE** | **EAF** | **Effect allele** | **Other allele** | **P-value** |
| --- | --- | --- | --- | --- | --- | --- |
| rs141108839 | 0.225336 | 0.0492624 | 0.0468092 | GT | G | 4.78E-06 |
| rs79120988 | 0.225337 | 0.0492624 | 0.0468094 | T | C | 4.78E-06 |
| rs75498960 | 0.225334 | 0.0492623 | 0.0468096 | G | A | 4.78E-06 |
| rs76643634 | 0.225333 | 0.0492622 | 0.0468098 | C | G | 4.78E-06 |
| rs79270640 | 0.225332 | 0.0492622 | 0.04681 | G | A | 4.78E-06 |
| rs78441633 | 0.225434 | 0.0492612 | 0.0468602 | T | C | 4.73E-06 |
| rs12563759 | 0.225329 | 0.049262 | 0.0468107 | C | T | 4.78E-06 |
| rs14330880,  rs202123707 | 0.225372 | 0.0492616 | 0.0468108 | ATTTTTCT | A | 4.76E-06 |
| rs78030635 | 0.225348 | 0.0492621 | 0.046811 | T | A | 4.77E-06 |
| rs17130694 | 0.225093 | 0.0492234 | 0.0469589 | T | C | 4.81E-06 |
| rs1225388371 | 2.62622 | 0.544068 | 0.000221035 | C | A | 1.39E-06 |
| rs543423023 | 1.42636 | 0.311997 | 0.000567497 | C | T | 4.84E-06 |
| rs150492835 | -0.675705 | 0.146232 | 0.00901904 | T | C | 3.82E-06 |
| rs533247012 | -0.603 | 0.127767 | 0.0112287 | G | A | 2.36E-06 |
| rs191684372 | -0.686046 | 0.143124 | 0.00946757 | T | A | 1.64E-06 |
| rs72981342 | -0.752125 | 0.16007 | 0.00762555 | T | C | 2.62E-06 |
| rs72989132 | -0.762143 | 0.162718 | 0.00741326 | A | C | 2.82E-06 |
| rs72976830 | -0.795587 | 0.164273 | 0.00742531 | C | T | 1.28E-06 |
| rs72976901 | -0.795478 | 0.164262 | 0.0074251 | T | C | 1.28E-06 |
| rs55960282 | -0.797046 | 0.164295 | 0.00742702 | T | C | 1.23E-06 |
| rs73082331 | -0.139848 | 0.0306299 | 0.161372 | A | G | 4.98E-06 |
| rs540605663 | -0.139753 | 0.03058 | 0.161968 | C | CAA | 4.88E-06 |
| rs201614713 | -0.139753 | 0.03058 | 0.161968 | C | CAGAGCG | 4.88E-06 |
| rs73082357 | -0.139624 | 0.0305805 | 0.161943 | A | G | 4.98E-06 |
| rs28497436 | 0.105372 | 0.0228169 | 0.349067 | C | T | 3.87E-06 |
| rs35333201 | 0.104853 | 0.0228032 | 0.350272 | C | T | 4.26E-06 |
| rs17017794 | 0.195693 | 0.0420266 | 0.0667623 | C | T | 3.22E-06 |
| rs568763284 | 1.28282 | 0.280431 | 0.000726519 | T | G | 4.77E-06 |
| rs73123505 | -0.145101 | 0.0293567 | 0.180542 | A | G | 7.71E-07 |
| rs67939396 | -0.145092 | 0.0293567 | 0.180542 | T | C | 7.72E-07 |
| rs1393875515 | 0.318543 | 0.0689315 | 0.0218738 | C | T | 3.82E-06 |
| rs184920366 | 0.337796 | 0.0739245 | 0.0194897 | A | G | 4.89E-06 |
| rs79335616 | 0.729765 | 0.153836 | 0.00342312 | T | C | 2.10E-06 |
| rs4739605 | 0.825826 | 0.177928 | 0.993352 | T | C | 3.46E-06 |
| rs80167526 | -0.27425 | 0.0598923 | 0.0409187 | G | T | 4.67E-06 |
| rs565744445 | 3.94945 | 0.854624 | 3.09E-05 | A | G | 3.81E-06 |
| rs759246692 | 1.53625 | 0.333407 | 0.000461814 | G | C | 4.07E-06 |
| rs72695283 | 0.119877 | 0.0259341 | 0.22618 | G | T | 3.79E-06 |
| rs12237383 | 0.119522 | 0.026155 | 0.220871 | A | G | 4.88E-06 |
| rs138993564 | 1.69058 | 0.368033 | 0.000404208 | G | A | 4.36E-06 |
| rs557968442 | 1.59946 | 0.347716 | 0.000416699 | G | A | 4.23E-06 |
| rs546620886 | 1.5946 | 0.347677 | 0.000418596 | T | C | 4.51E-06 |
| rs554043934 | 1.5933 | 0.348693 | 0.000423758 | C | A | 4.89E-06 |
| rs71376889 | 0.150616 | 0.0327168 | 0.858143 | C | CTG | 4.15E-06 |
| rs569593373 | 0.541218 | 0.112894 | 0.00648127 | T | C | 1.63E-06 |
| rs187881984 | 0.54174 | 0.112157 | 0.00659088 | G | C | 1.36E-06 |
| rs573888121 | 0.53122 | 0.112569 | 0.00658682 | C | T | 2.37E-06 |
| rs193037486 | 0.504294 | 0.109113 | 0.00709981 | A | G | 3.80E-06 |
| rs17110702 | -0.167265 | 0.0363653 | 0.110304 | T | C | 4.23E-06 |
| rs7098930 | -0.161442 | 0.0345884 | 0.123674 | A | G | 3.05E-06 |
| rs4918926 | -0.158463 | 0.034603 | 0.123321 | T | C | 4.66E-06 |
| rs4918927 | -0.169604 | 0.0364378 | 0.110443 | G | A | 3.25E-06 |
| rs11188312 | -0.14659 | 0.0315612 | 0.152076 | T | G | 3.41E-06 |
| rs10882591 | -0.197625 | 0.0402223 | 0.0905894 | T | C | 8.95E-07 |
| rs187634809 | 2.10475 | 0.453546 | 0.000227038 | T | C | 3.47E-06 |
| rs73220964 | 0.302751 | 0.0642442 | 0.0262834 | T | A | 2.45E-06 |
| rs3024577 | -0.103926 | 0.0226321 | 0.396668 | G | A | 4.39E-06 |
| rs3024585 | -0.103619 | 0.0226141 | 0.396683 | A | G | 4.60E-06 |
| rs34459436 | 0.108601 | 0.0237771 | 0.391898 | A | G | 4.94E-06 |
| rs567607900 | 1.42882 | 0.304209 | 0.000584267 | T | C | 2.64E-06 |
| rs371728287 | -1.07128 | 0.225144 | 0.00493203 | T | C | 1.95E-06 |
| rs78674086 | -0.386927 | 0.084655 | 0.0226459 | G | A | 4.86E-06 |
| rs188850959 | -0.651328 | 0.142135 | 0.00886046 | G | T | 4.60E-06 |
| rs146248039 | -1.71589 | 0.35753 | 0.00279343 | A | C | 1.59E-06 |

SNP, single nucleotide polymorphism; SE, standard error; EAF, effect allele frequency; Beta coefficients are in standard deviation (SD) unit per allele; effect allele is the broad depression-raising allele.

*<http://www.finngen.fi/en>

1. Otowa T, Hek K, Lee M, et al (2016) Meta-analysis of genome-wide association studies of anxiety disorders. Mol Psychiatry 21:1391–1399. <https://doi.org/10.1038/mp.2015.197>

2. Forstner AJ, Awasthi S, Wolf C, et al (2021) Genome-wide association study of panic disorder reveals genetic overlap with neuroticism and depression. Mol Psychiatry 26:4179–4190. https://doi.org/10.1038/s41380-019-0590-2

3. Howard DM, Adams MJ, Clarke T-K, et al (2019) Genome-wide meta-analysis of depression identifies 102 independent variants and highlights the importance of the prefrontal brain regions. Nat Neurosci 22:343–352. <https://doi.org/10.1038/s41593-018-0326-7>

4. Nagel M, Watanabe K, Stringer S, et al (2018) Item-level analyses reveal genetic heterogeneity in neuroticism. Nat Commun 9:905. https://doi.org/10.1038/s41467-018-03242-8

5. ADHD Working Group of the Psychiatric Genomics Consortium (PGC), Early Lifecourse & Genetic Epidemiology (EAGLE) Consortium, 23andMe Research Team, et al (2019) Discovery of the first genome-wide significant risk loci for attention deficit/hyperactivity disorder. Nat Genet 51:63–75. https://doi.org/10.1038/s41588-018-0269-7

6. Autism Spectrum Disorder Working Group of the Psychiatric Genomics Consortium, BUPGEN, Major Depressive Disorder Working Group of the Psychiatric Genomics Consortium, et al (2019) Identification of common genetic risk variants for autism spectrum disorder. Nat Genet 51:431–444. https://doi.org/10.1038/s41588-019-0344-8

7. eQTLGen Consortium, BIOS Consortium, the Bipolar Disorder Working Group of the Psychiatric Genomics Consortium, et al (2019) Genome-wide association study identifies 30 loci associated with bipolar disorder. Nat Genet 51:793–803. https://doi.org/10.1038/s41588-019-0397-8

8. Trubetskoy V, Pardiñas AF, Qi T, et al (2022) Mapping genomic loci implicates genes and synaptic biology in schizophrenia. Nature 604:502–508. https://doi.org/10.1038/s41586-022-04434-5

# Supplementary Figures


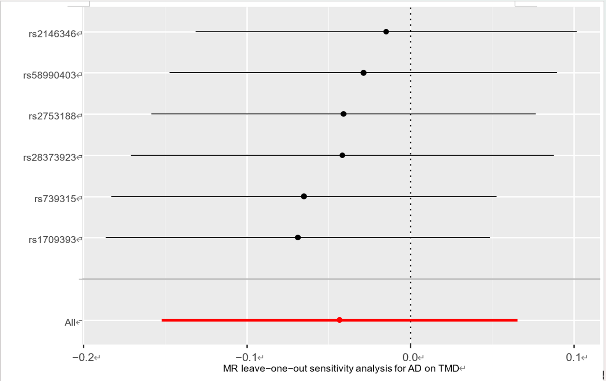

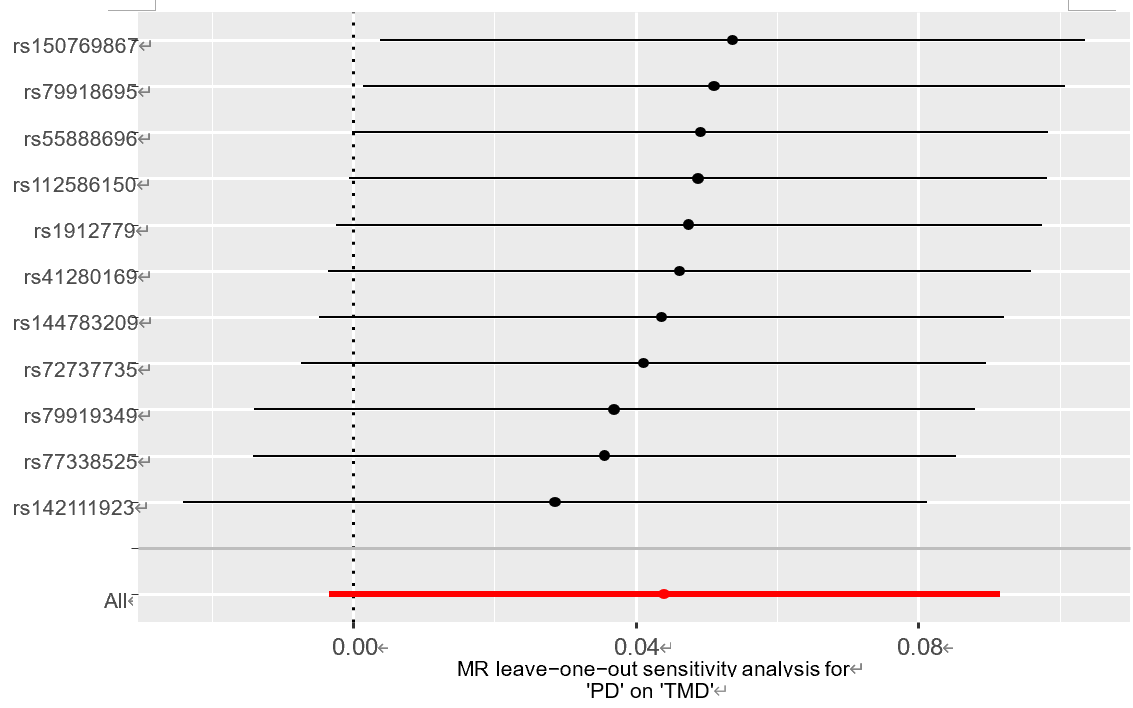


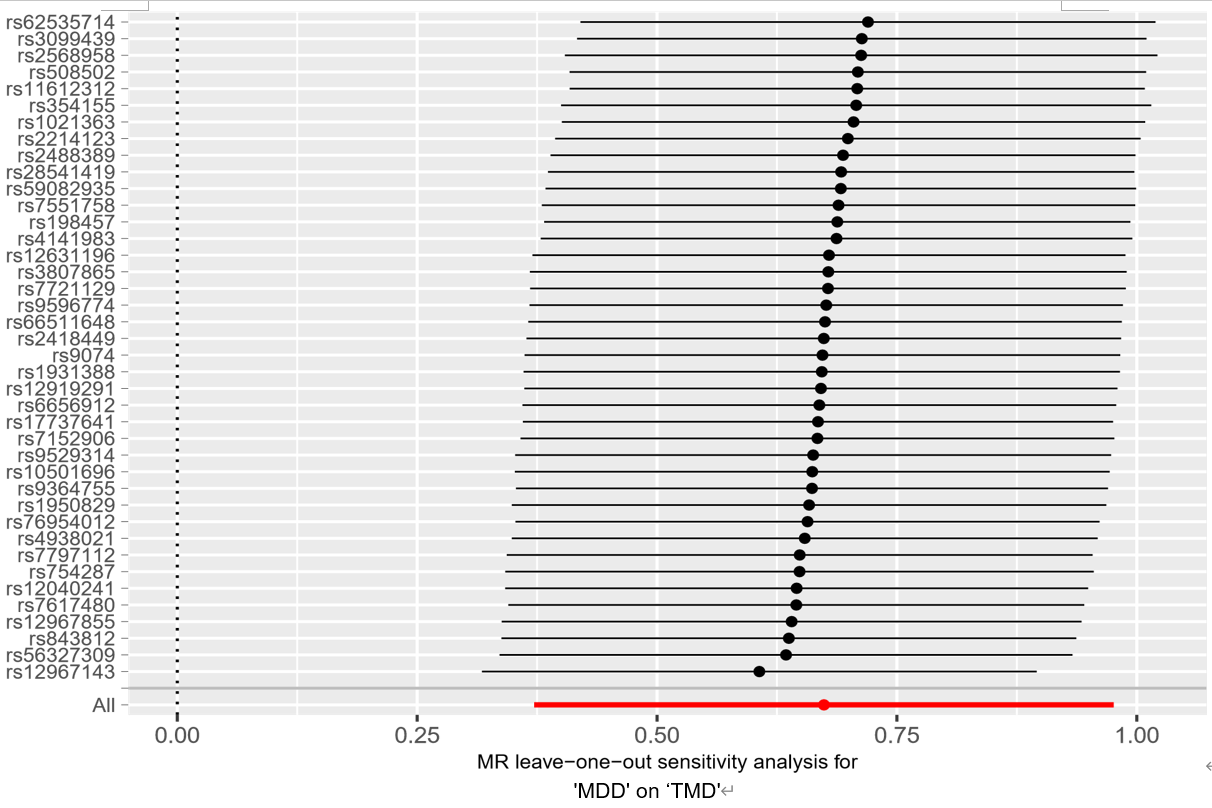

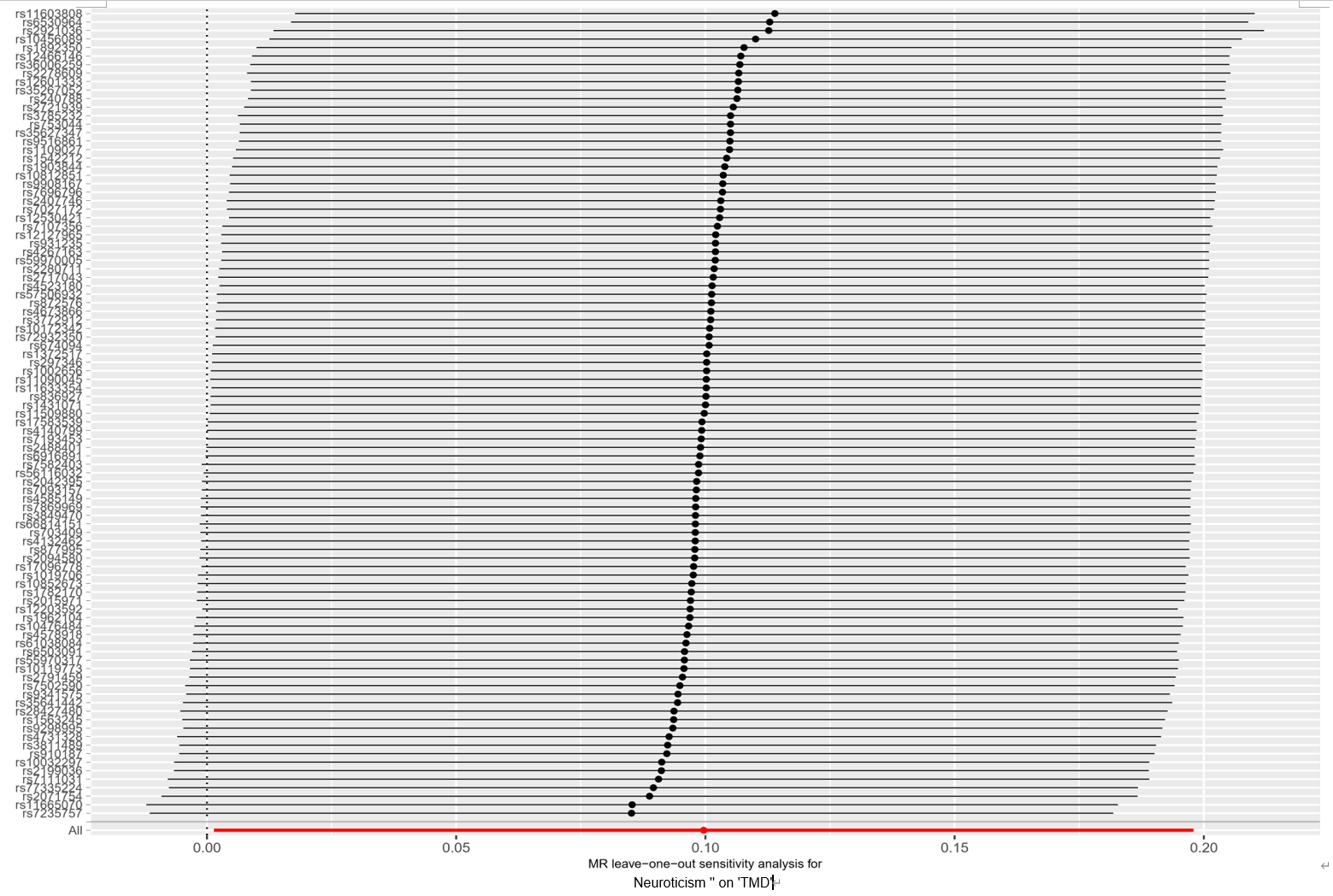


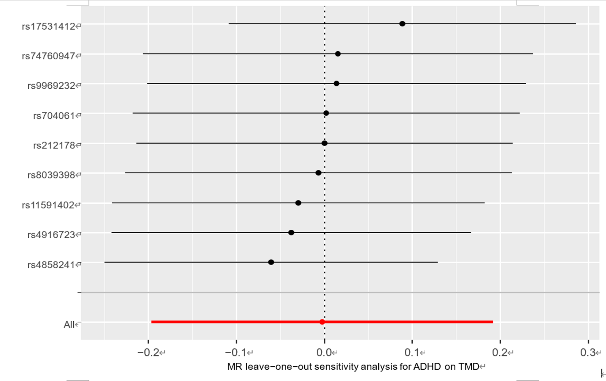

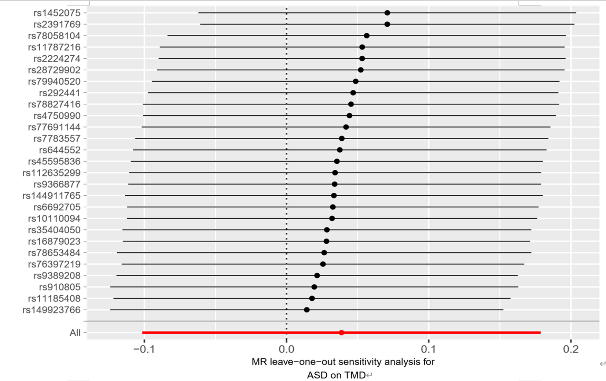


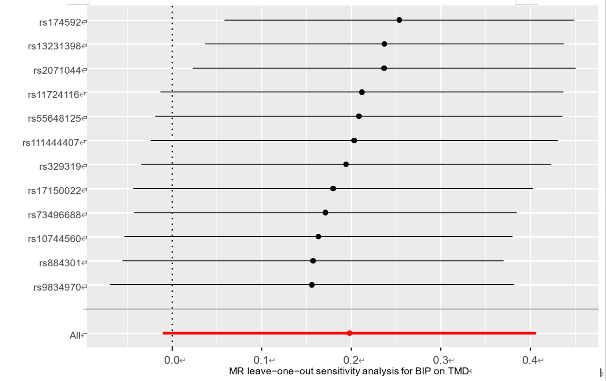

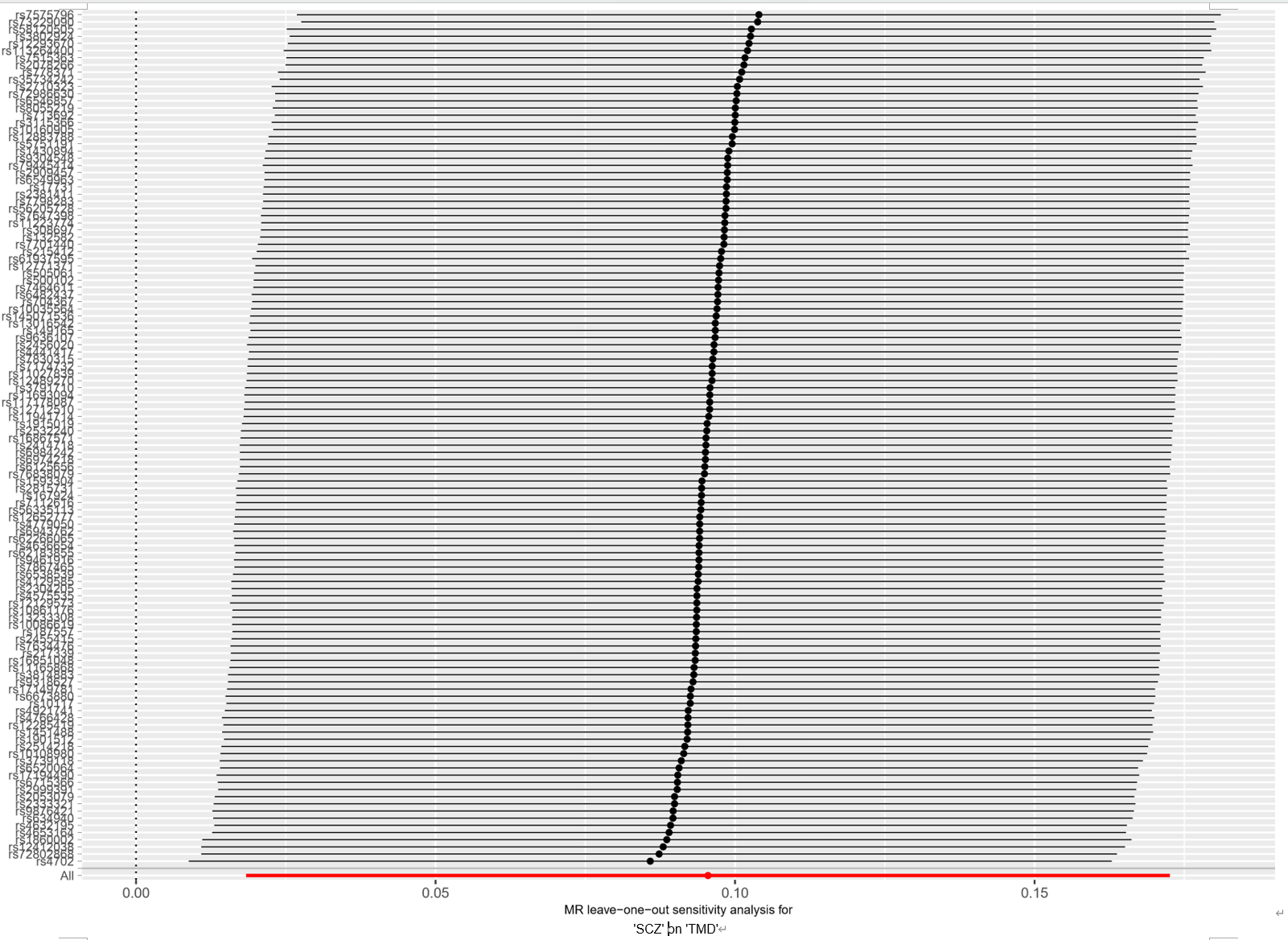


**Figure S1.** Leave-one-out analysis of associations of eight psychiatric traits on the risk of temporomandibular disorders.

TMD, temporomandibular disorders; AD, anxiety disorder; PD, panic disorder; MDD, major depressive disorder; ADHD, attention deficit hyperactivity disorder; ASD, autism spectrum disorder; BIP, bipolar disorder; SCZ, schizophrenia.


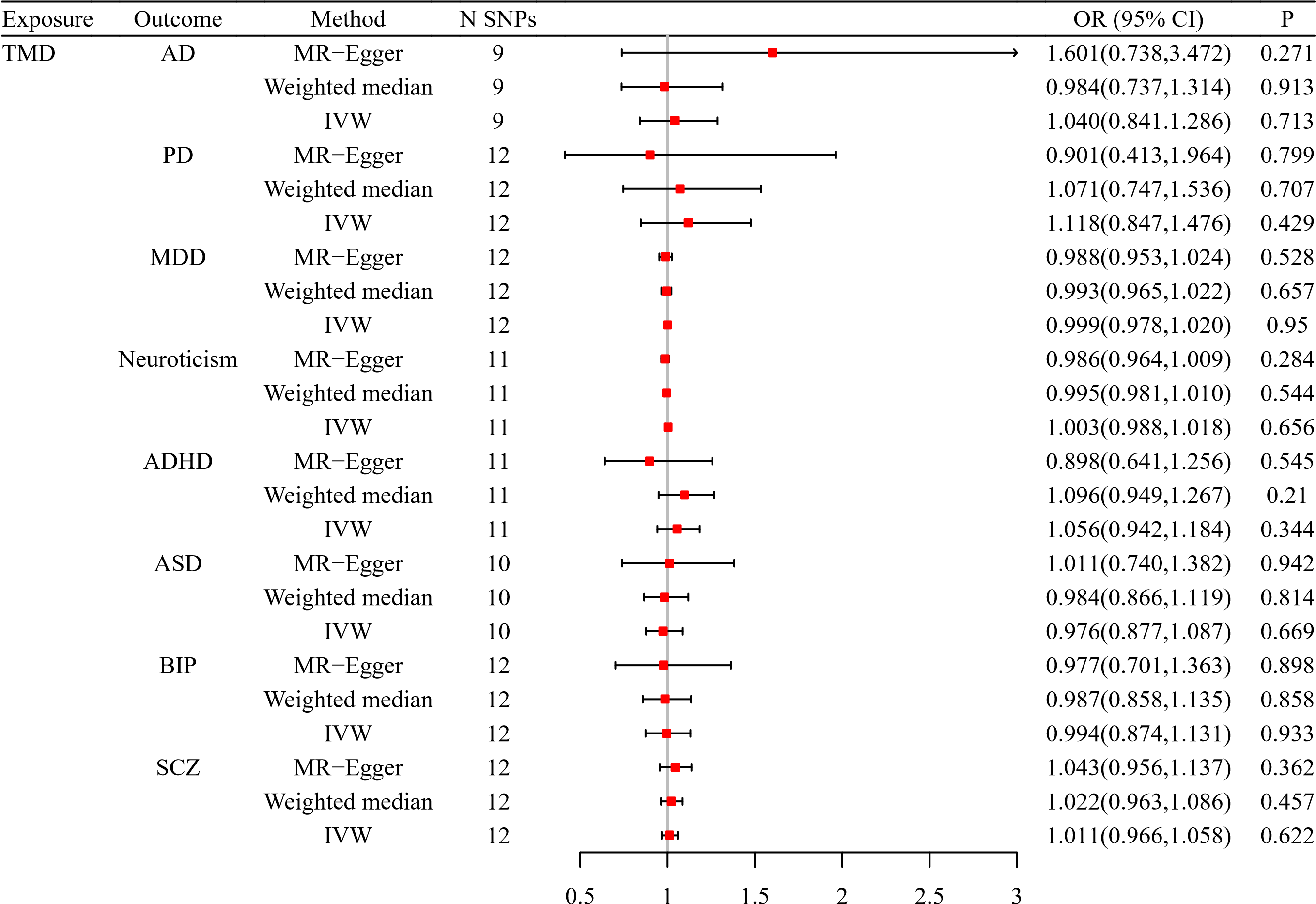


**Figure S2. Two-sample Mendelian randomization analyses showing the effect estimates of TMD on eight psychiatric traits.**

IVW, inverse-variance weighted; MR, Mendelian randomization; N SNPs, number of the SNPs used in MR analysis; OR, odds ratio; CI, confidence interval; TMD, temporomandibular disorders; AD, anxiety disorder; PD, panic disorder; MDD, major depressive disorder; ADHD, attention deficit hyperactivity disorder; ASD, autism spectrum disorder; BIP, bipolar disorder; SCZ, schizophrenia.
